# Interpretation of genome-wide infinium methylation data from ligated DNA in formalin-fixed, paraffin-embedded paired tumor and normal tissue

**DOI:** 10.1186/1756-0500-5-117

**Published:** 2012-02-22

**Authors:** Farzana Jasmine, Ronald Rahaman, Shantanu Roy, Maruf Raza, Rupash Paul, Muhammad Rakibuz-Zaman, Rachelle Paul-Brutus, Charlotte Dodsworth, Mohammed Kamal, Habibul Ahsan, Muhammad G Kibriya

**Affiliations:** 1 Department of Health Studies, The University of Chicago, Chicago, IL 60637, USA; 2 Department of Human Genetics, The University of Chicago, Chicago, IL 60637, USA; 3 Department of Medicine, The University of Chicago, Chicago, IL 60637, USA; 4 Department of Comprehensive Cancer Center, The University of Chicago, Chicago, IL 60637, USA; 5 University of Chicago Research Office in Bangladesh, Dhaka, Bangladesh; 6 Department of Pathology, Bangabandhu Sheikh Mujib Medical University (BSMMU), Dhaka 1000, Bangladesh

## Abstract

**Background:**

Formalin-fixed, paraffin-embedded (FFPE) samples are a highly desirable resource for epigenetic studies, but there is no suitable platform to assay genome-wide methylation in these widely available resources. Recently, Thirlwell et al. (2010) have reported a modified ligation-based DNA repair protocol to prepare FFPE DNA for the Infinium methylation assay. In this study, we have tested the accuracy of methylation data obtained with this modification by comparing paired fresh-frozen (FF) and FFPE colon tissue (normal and tumor) from colorectal cancer patients. We report locus-specific correlation and concordance of tumor-specific differentially methylated loci (DML), both of which were not previously assessed.

**Methods:**

We used Illumina's Infinium Methylation 27K chip for 12 pairs of FF and 12 pairs of FFPE tissue from tumor and surrounding healthy tissue from the resected colon of the same individual, after repairing the FFPE DNA using Thirlwell's modified protocol.

**Results:**

For both tumor and normal tissue, overall correlation of β values between all loci in paired FF and FFPE was comparable to previous studies. Tissue storage type (FF or FFPE) was found to be the most significant source of variation rather than tissue type (normal or tumor). We found a large number of DML between FF and FFPE DNA. Using ANOVA, we also identified DML in tumor compared to normal tissue in both FF and FFPE samples, and out of the top 50 loci in both groups only 7 were common, indicating poor concordance. Likewise, while looking at the correlation of individual loci between FFPE and FF across the patients, less than 10% of loci showed strong correlation (r ≥ 0.6). Finally, we checked the effect of the ligation-based modification on the Infinium chemistry for SNP genotyping on an independent set of samples, which also showed poor performance.

**Conclusion:**

Ligation of FFPE DNA prior to the Infinium genome-wide methylation assay may detect a reasonable number of loci, but the numbers of detected loci are much fewer than in FF samples. More importantly, the concordance of DML detected between FF and FFPE DNA is suboptimal, and DML from FFPE tissues should be interpreted with great caution.

## Background

Aberrant DNA methylation is a well-established pathway in carcinogenesis [1,2]. In colorectal cancer (CRC), global hypomethylation of DNA and gene-specific hypermethylation of tumor suppressor genes and microRNA genes are extensively studied [3]. For example, seminal work in cancer epigenetics has shown that most cases of microsatellite-instable CRC are caused by the hypermethylation and consequent silencing of the mismatch-repair gene *MLH1 *[4,5]. Many epigenetic markers for CRC are now known, including *MGMT *[6,7], *VIM *[8], *APC *[9], *RUNX3 *[5,10], *CDKN2A *[11], and numerous others found in recent genome-wide studies [12-14]. It is hoped that continuing studies can provide useful strategies for detection, treatment, and the understanding of etiology [15].

Formalin-fixed, paraffin-embedded (FFPE) samples are routinely collected for histopathological diagnosis and are thus a highly desirable resource for epigenetic studies. Though formalin fixation does not alter the methylation status of cytosine [16], it does cause other forms of DNA damage, including cross-linking, fragmentation, and generation of apurinic/apyrimidinic sites [17]. This degradation can be detrimental to qPCR [18] or whole-genome amplification (WGA) [19], which are integral steps in many methylation assays.

Therefore, any existing methylation assay must be carefully evaluated before it can be confidently used for FFPE-derived DNA. Many methylation assays have been evaluated for such purposes [20-26]. The most comprehensive validations involve comparisons between paired FFPE and fresh-frozen (FF) tissue samples, such as the validations reported for: high-resolution melting analysis [27], qPCR quantification after methylation-specific restriction enzyme digestion [20], bisulfite sequencing [21], and Illumina's GoldenGate methylation assay [23]. Killian's validation of the GoldenGate assay showed good correlation between paired FFPE and FF samples but the GoldenGate assay interrogates a limited number of CpG loci and is not suitable for studies of large numbers of loci [23]. For genome-wide studies, Illumina's Infinium assay [28] allows thousands of loci to be interrogated at a time. However, the Infinium chemistry depends on WGA and thus was originally designed for high-quality, high molecular weight DNA. Many WGA protocols fail with fragmented FFPE DNA [19], and Illumina's proprietary Infinium WGA chemistry has been shown to fail with these samples by both in-house [29] and independent results [30]. GoldenGate chemistry, on the other hand, does not involve WGA, targets small fragments of DNA for PCR amplification, and thus may be compatible with FFPE DNA. In a previous study we compared genome-wide gene expression (using Illumina's DASL assay, based on GoldenGate chemistry) in paired FF and FFPE breast tumor tissues and surrounding healthy tissues [31]. In that study, we found that the tumor specific differentially expressed genes detected in FF and FFPE samples were significantly different, suggesting that interpreting FFPE gene expression data may be problematic.

Recently, Thirlwell et al. described a modified Infinium methylation protocol in which FFPE DNA was repaired by ligation prior to the bisulfite conversion and methylation assay [30]. Thirlwell's protocol was shown to be effective in several respects. First, the authors showed that ligation allowed successful WGA, whereas unligated replicates failed to amplify. Second, the authors demonstrated reasonable correlation between paired FF and FFPE samples from primary ovarian cancer tissues. However, they did not report whether FFPE tumor samples could detect the same differentially methylated loci (DML) as were detected by examining FF tumor tissue. Recently, we reported results from a genome-wide DNA methylation study in colorectal cancer using Illumina's Infinium-based HumanMethylation27 microarray [12]. The study was conducted using paired FF tumor and adjacent normal colon tissue samples from 24 patients. FFPE tumor and normal samples were also available from the same patients. This provided an excellent opportunity to independently test the utility of ligated-FFPE DNA for Infinium methylation analysis using Thirlwell's modification [30]. In the current study, we have tested the accuracy of methylation data from ligated-FFPE DNA through numerous correlations with paired FF DNA for both CRC and adjacent normal colon tissue. We also identified DML in FF tumor DNA (compared to FF adjacent normal) and compared these to loci that were differentially methylated in ligated-FFPE tumor DNA (compared to ligated-FFPE adjacent normal) from the same patients. Our study is unique amongst other validations of FFPE methylation assays in that we have reported results for locus-specific correlations across samples and concordance of tumor-specific DML.

## Methods

### Tissue samples

Colon tissues (tumor and surrounding healthy) were collected from surgically removed colonic segments from consecutive patients at Bangabandhu Sheikh Mujib Medical University (BSMMU), Dhaka, Bangladesh, as described previously [12]. All samples were collected by one surgical pathology fellow (MR) from the operating room immediately after surgical resection during the period of December 2009 to March 2010. Histopathology was done independently by two histopathologists (MK & MR), and there was concordance in all cases. For each patient, one sample was collected from the tumor mass, and another sample was taken from the resected, unaffected part of the colon about 5-10 cm away from the tumor mass. From each site, tissue sections were preserved as (1) fresh frozen, (2) in RNA-stabilizing buffer and (3) as FFPE block. The samples were shipped on dry ice to the molecular genomics lab at the University of Chicago for subsequent DNA extraction and methylation assay. We also received the corresponding FFPE blocks that were used for histopathology. Written informed consent was obtained from all participants. The research protocol was approved by the "Ethical Review Committee, Bangabandhu Sheikh Mujib Medical University", Dhaka, Bangladesh (BSMMU/2010/10096) and by the "Biological Sciences Division, University of Chicago Hospital Institutional Review Board", Chicago, IL, USA (10-264-E). We have previously reported genome-wide methylation data from the first 24 paired (tumor and corresponding healthy colonic tissue) FF DNA [12]. In this paper we present methylation data from FFPE sections of the first 12 consecutive patients of the same series for whom we had paired (normal and CRC) FFPE blocks available and compared the data with corresponding 12 pairs from DNA from FF samples.

### DNA extraction and quality control

DNA was extracted from FFPE tissue (tumor and surrounding healthy tissue) using the Puregene Core kit A (Qiagen, Maryland, USA). During extraction all DNA samples were treated with RNase. FFPE tissues were about 1 year old. All DNA concentrations were measured by Nanodrop (Thermo-Fisher, USA), and integrity was checked by the Agilent Bioanalyzer 2100 using the DNA 12000 kit (Agilent Technologies, USA).

### Bisulfite conversion

2 μg FFPE DNA were ligated before starting bisulfite conversion using the protocol described by Thirlwell et al. [30]. For bisulfite conversion, the EZ DNA methylation kit (Zymo Research, USA) was used.

### Genome-wide methylation assay

The Infinium Methylation assay (Illumina Inc., USA) was done using the Methylation 27K chip, which contains 27,578 CpG sites spanning 14,495 genes. The CpG sites were located within the proximal promoter regions of genes, with the distance to transcription start site (TSS) ranging from 0 to 1499 bp and averaged at 389 ± 341 bp. Paired FFPE DNA from CRC and surrounding normal colonic tissues were processed on the same chip to avoid batch effects, and all 24 FFPE samples were processed on 2 chips (12 samples per chip). It may be noted that the corresponding 24 FF samples were processed in a different batch previously, but the corresponding DNA samples from normal and CRC tissue were processed in the same chip. A Tecan Evo robot was used for automated sample processing and the chips were scanned on a single BeadArray reader (S428). Illumina's BeadStudio analytical software showed excellent intensity for staining (above 15000), clear clustering for the hybridization probes, good target removal intensity (< 400) and satisfactory bisulfite conversion.

### Genome-wide methylation data analysis

For measuring methylation, we used the Illumina BeadStudio software to generate the β value for each locus from the intensity of methylated and unmethylated probes. The β value is calculated as (intensity of methylated probe)/(intensity of methylated probe + intensity of unmethylated probe). Hence, β ranges between 0 (least methylated) and 1 (most methylated) and is proportional to the degree of the methylated state of a particular loci. The methylation module of BeadStudio was used for differential methylation analysis using Illumina's custom model. The model operates under the assumption that the methylation value β is normally distributed among biological replicates corresponding to a set of biological conditions (tumor and normal in the present scenario). The DiffScore of a probe is computed as:

$$\text{DiffScore}=10\;\text{Sign}\left(\beta_{\text{tumor}}-\beta_{\text{normal}}\right)\log_{10}\text{p}$$

where p represents the p-value from *t*-test.

$$\text{Delta}\beta =\left(\beta_{\text{tumor}}-\beta_{\text{normal}}\right)$$

In addition to the BeadStudio differential methylation analysis, we exported the BeadStudio generated β values to the PARTEK Genomic Suite [32] for further statistical analyses. Principal component analysis (PCA) and sample histograms were checked as a part of quality control analyses of the data. Mixed-model multi-way ANOVA (which allows more than one ANOVA factor to be entered in each model) was used to compare the individual CpG loci methylation data across different groups. Two of the FFPE samples were excluded from the analysis due to poor gene detection. The remaining analyses were done with 10 pairs of FF and FFPE tissue. In general, tissue type (tumor or adjacent normal), sex (male or female) and tumor location (proximal colon or distal colon) were used as categorical variables with fixed effect since these represent all conditions of interest; whereas "case ID#" (used as a proxy of inter-person variation) was treated as a categorical variable with random effect, since the person ID is only a random sample of all the levels of that factor. Method of moments estimation was used to obtain estimates of variance components for mixed models [33]. In the ANOVA model, the β value for a locus was used as the response variable, and tissue type (tumor or normal), age category (≤ 40 yrs vs. > 40 yrs), case ID#, sex and location were entered as ANOVA factors. It may be noted that age category, sex and location were nested within case ID#. One example of a model is as follows:

$${\text{Y}}_{\text{ijklmn}}=\mu +{\text{Tissue}}_{\text{i}}+\text{Age}\_\text{Cat}40_{\text{j}}+{\text{Sex}}_{\text{k}}+{\text{Location}}_{1}+\text{Person}{\left(\text{Age}\_\text{Cat}40*\text{Sex}*\text{Location}\right)}_{\text{jklm}}+\varepsilon_{\text{ijklmn}}$$

Where Y_ijklmn _represents the n^th ^observation on the i^th ^Tissue, j^th ^Age_Cat40, k^th ^Sex, l^th ^Location and m^th ^Person; μ is the common effect for the whole experiment, ε_ijklmn _represents the random error present in the n^th ^observation on the i^th ^Tissue, j^th ^Age_Cat40, k^th ^Sex, l^th ^Location, and m^th ^Person. The errors ε_ijklmn _are assumed to be normally and independently distributed with mean 0 and standard deviation δ for all measurements. An FDR of 0.05 was used for multiple testing correction.

The correlation of β values between FF and FFPE samples from each individual was checked in both normal and tumor tissue. Then the correlation of the average β values of all the FF and corresponding FFPE samples was analyzed both for normal and tumor tissue.

The distribution of the genes correlated between FF and FFPE samples were also checked. The top 50 differentially methylated genes between normal and tumor tissue in both FF and FFPE tissue were detected to find the common genes.

## Results

### Detection of loci

**Figure 1:**
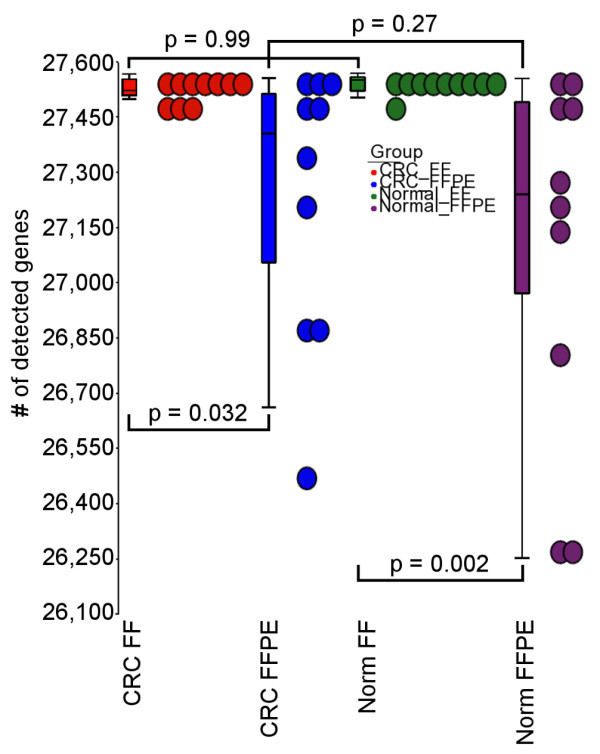
**Number of loci detected in each sample group**. The sample groups shown are: tumor FF tissue (red, average detected loci = 27530.6 ± 27.4); tumor FFPE tissue (blue, 27,239.0 ± 375.4), normal FF tissue (green, 27,541.1 ± 26.7), and normal FF tissue (violet, 27,102.5 ± 502.0). There was significant difference between average detected loci in normal FF vs. normal FFPE (*p *= 0.002) and between tumor FF vs. tumor FFPE (*p *= 0.032).

In the microarray, a locus was said to be detected if the average signal intensity of that locus was significantly (p < 0.05) higher than the built-in negative control on the chip. In terms of the number of detected loci per sample, there was no statistical difference between normal and tumor DNA from FF tissue. However, in FFPE DNA, for both normal and tumor tissues, the number of detected loci per sample was significantly lower than in FF DNA (Figure 1), although there was no difference in the number of detected loci between normal and tumor within FFPE blocks. The histogram showing the distribution of signal intensity is presented in Additional file 1: Figure S1. The data discussed in the publication is deposited in NCBI's Gene Expression Omnibus and will be accessible through GEO (accession #GSE33181).

### Sources of variation in methylation

**Figure 2:**
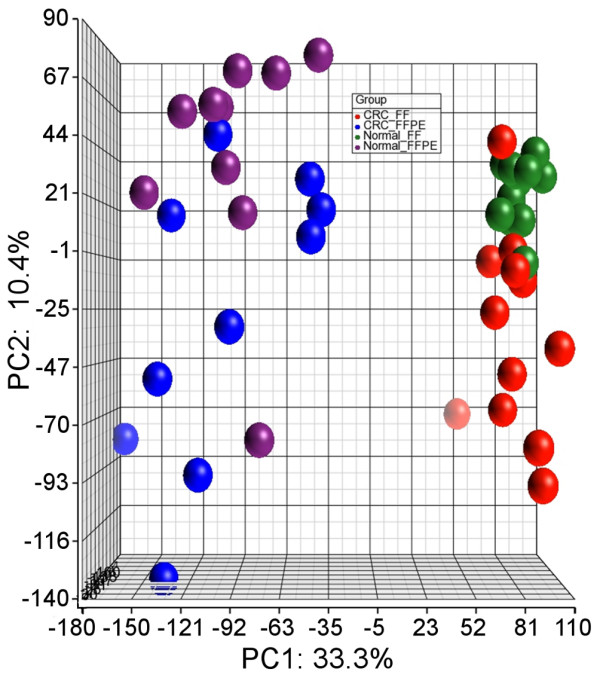
**Principle components analysis, displaying spatial separation of: FF tumor tissue (red), FF normal tissue (green), FFPE tumor tissue (blue), and FFPE normal tissue (purple)**. The color coding suggests that the separation along the horizontal axis (PC1) may be attributed to storage type (FF vs. FFPE), and the separation along the vertical axis (PC2) may be attributed to tissue type (tumor vs. normal).

Principal components analysis (Figure 2) shows that the samples cluster by both storage type (FF vs. FFPE) and tissue type (tumor vs. normal). There is much greater separation between samples of different storage type (PC1 = 33.3%, shown on the x-axis) than between samples of different tissue type (PC2 = 10.4%, shown on the y-axis). It is also notable that, for FFPE tissue, the clustering of tumor and normal samples shows poorer separation than the clustering of tumor and normal samples in FF tissue.

### Histograms of methylation β-values by storage type

**Figure 3:**
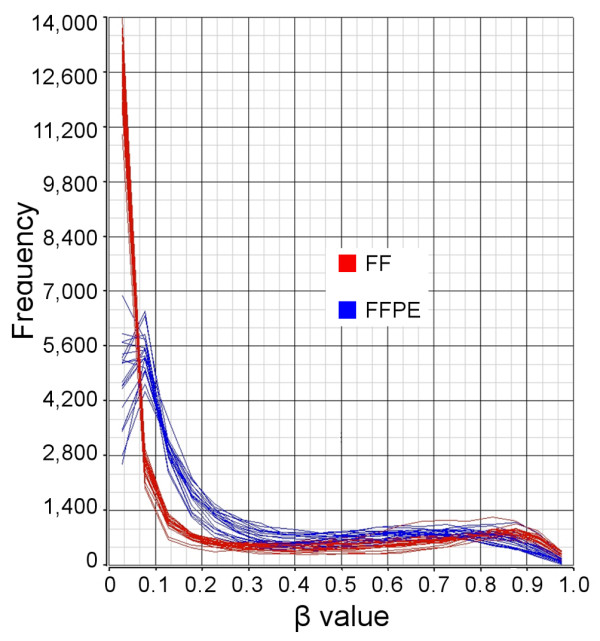
**Histograms of methylation β-values by storage type**. The histograms show the frequency distribution of 26,486 autosomal loci in FF samples (red) or FFPE samples (blue), where each line represents one sample.

The distribution of β values for FF (Figure 3, shown in red) and FFPE samples (shown in blue) clearly shows that the vast majority of loci were hypomethylated (below 0.15) and only a few were hypermethylated (above 0.5) in all samples. However, the FFPE samples clustered slightly separately than FF samples.

### Correlation between storage-type and tissue-type pairs

**Figure 4:**
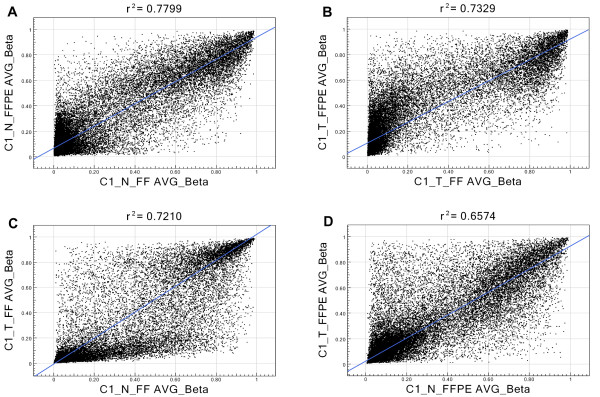
**Scatter plots of β-values for sample pairs from patient C1**. Each point represents the β value of one locus in this patient. For each plot, the straight line is the regression line and r^2 ^is Pearson's correlation coefficient squared. Top: Correlation between paired FF and FFPE samples for (**A**) normal colon tissue and (**B**) tumor tissue. Bottom: Correlation between paired tumor and normal samples for (**C**) FF tissue and (**D**) FFPE tissue.

β values of all 27,578 loci from each FFPE sample were plotted against the β values from corresponding FF tissue of the same patient. Representative scatter plots from one patient (C_1) are shown in Figure 4A (for normal tissue) and 4B (for tumor tissue), where each dot represents a locus in a single sample. Overall the degree of scatter suggests that, at the individual patient level, the methylation status in FFPE tissue correlated poorly with paired FF tissue. This poor correlation may be explained by the DNA damage from formalin fixation. It may be noted that we have seen technical replicates to show very tight correlation (r^2 ^> 0.99) in the Infinium methylation assay (see Additional file 2: Figure S2).

Then the β values of all 27,578 loci from each tumor sample were plotted against the β values from the corresponding normal tissue of the same patient. Representative scatter plots from one patient (C_1) are shown in Figure 4C (for FF) and 4D (for FFPE). As expected, the scatter plots indicate differential methylation of a number of loci in CRC tissue compared to normal tissue.

**Figure 5:**
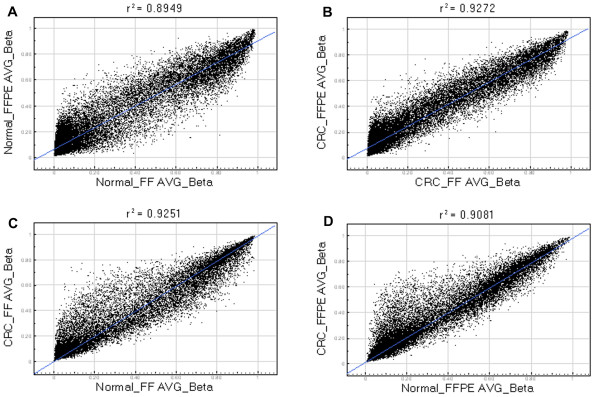
**Scatter plots of average β values for all sample pairs**. Each point represents the β value of one locus averaged over all 10 patients. (**A**) Average β values of normal FFPE tissue vs. average β values of normal FF tissue. (**B**) Average β values of tumor FFPE tissue vs. average β values of tumor FF tissue. (**C**) Average β values of tumor FF tissue vs. average β values of normal FF tissue. (**D**) Average β values of tumor FFPE tissue vs. average β values of normal FFPE tissue.

In the next step, instead of using β values from individual patients, we used the mean β values from all 10 patients and plotted the values from FFPE against FF. Scatter plots showed that the correlation calculated in the total samples was better (r^2 ^= 0.89 for normal tissue and r^2 ^= 0.93 for tumor tissue) than the correlation that was seen at the individual sample level (Figure 5A and 5B). Even then, the scatter suggests that many loci do not correlate well between FF and FFPE.

Similarly, we looked at the correlation of mean β values of tumor FF tissue vs. mean β-values of normal FF tissue (r^2 ^= 0.93, Figure 5C); and mean β values of tumor FFPE tissue vs. mean β values of normal FFPE tissue (r^2 ^= 0.91, Figure 5D). Average tumor vs. normal β values showed better correlation than individual sample pairs, for both FF and FFPE tissue. This increase in r^2 ^is related to the direct increase in data points used for analysis.

### Locus-specific correlation between FF and FFPE DNA

**Figure 6:**
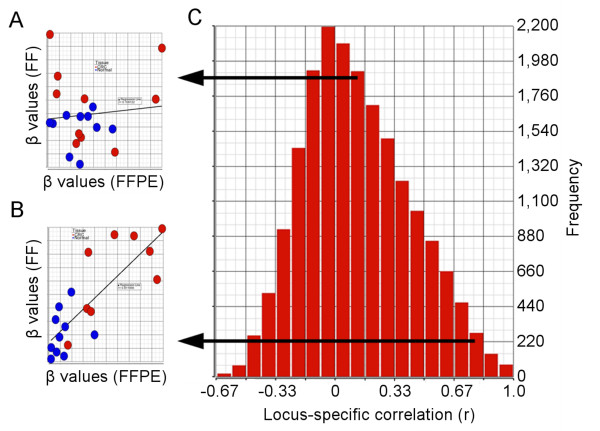
**Correlation between FF and FFPE β values for individual methylation loci**. For (**A**) and (**B**), each data point represents a single sample for which the β value of the FFPE is shown on the x-axis and the corresponding β value from FF is shown on the y-axis. Red points are tumor samples, and blue points are from normal samples. (**A**) A representative locus for which correlation between FF and FFPE samples was poor. (**B**) A representative locus for which correlation between FF and FFPE samples was good. (**C**) Histogram showing the distribution of resultant r values from FF/FFPE correlations for all loci.

Methylation is significantly different between males and females, mainly due to the sex chromosomal loci [12]; therefore we excluded the sex chromosomal loci from subsequent analyses. For each of the 26,486 autosomal loci, we correlated β values of FF and FFPE in all 20 paired samples (Figure 6). Representative scatter plots for two individual loci are shown in Figure 6A and 6B, where each dot represents a sample. The distribution of the resultant r values for all 26,486 loci are shown in the histogram in Figure 6C. The figure clearly shows that less than 10% of the loci correlated well in FF and FFPE samples (only 2,463 loci had r ≥ 0.6), and the vast majority of the loci showed poor correlation between corresponding FF and FFPE tissue. Therefore, our data suggests that the use and interpretation of the correlation analyses requires caution, especially when considering a large number of loci per sample.

### Differentially methylated loci (DML) in FFPE compared to FF

**Figure 7:**
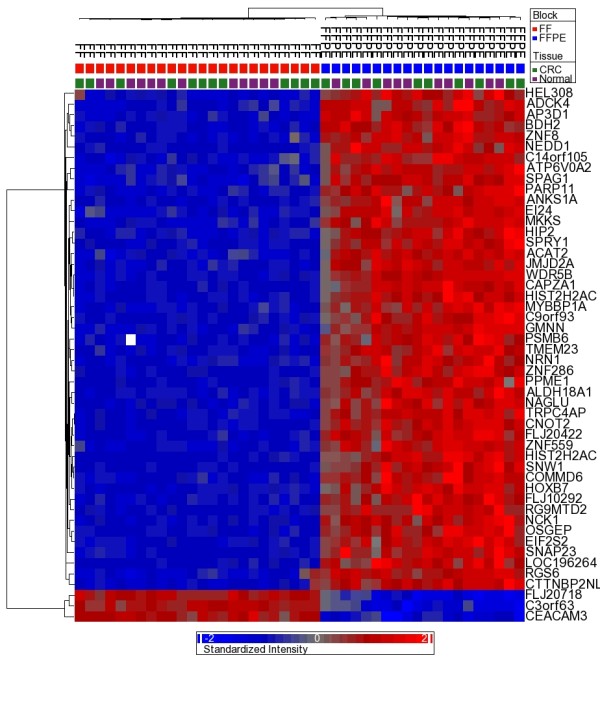
**Unsupervised clustering and heatmap based on the top 50 DML that differentiate FF and FFPE samples in combined analysis of all samples studied**.

In a paired *t*-test, there were a total of 17,896 autosomal loci (67.56%) that were significantly differentially methylated at FDR 0.05; of these, the absolute Δβ was greater than 0.2 for 922 loci. In an unpaired *t*-test with bootstrapping, 9,475 autosomal loci (35.77%) were differentially methylated (bootstrap ≤ 0.05); of these, the absolute Δβ was greater than 0.2 in 652 loci. We also used a multivariate ANOVA model that controls for tissue type (tumor vs. normal), person-to-person variation, age category (above or below 40 yrs), sex, tumor location (proximal vs. distal); this analysis also revealed differential methylation in a total of 18,660 loci (70.45%) at FDR 0.05 level that were differentially methylated in FFPE tissue compared to corresponding FF tissue. Of these, the absolute Δβ was greater than 0.2 in 914 loci. Overlap between these analyses is presented in Additional file 3: Figure S3, which clearly indicates that regardless of which statistical test is applied, there are a large number of loci that show significant differential methylation in FFPE samples compared to corresponding FF sample. Unsupervised clustering using these loci can very effectively differentiate FFPE samples from FF samples (Figure 7).

### Differentially methylated loci in tumor tissue

**Figure 8:**
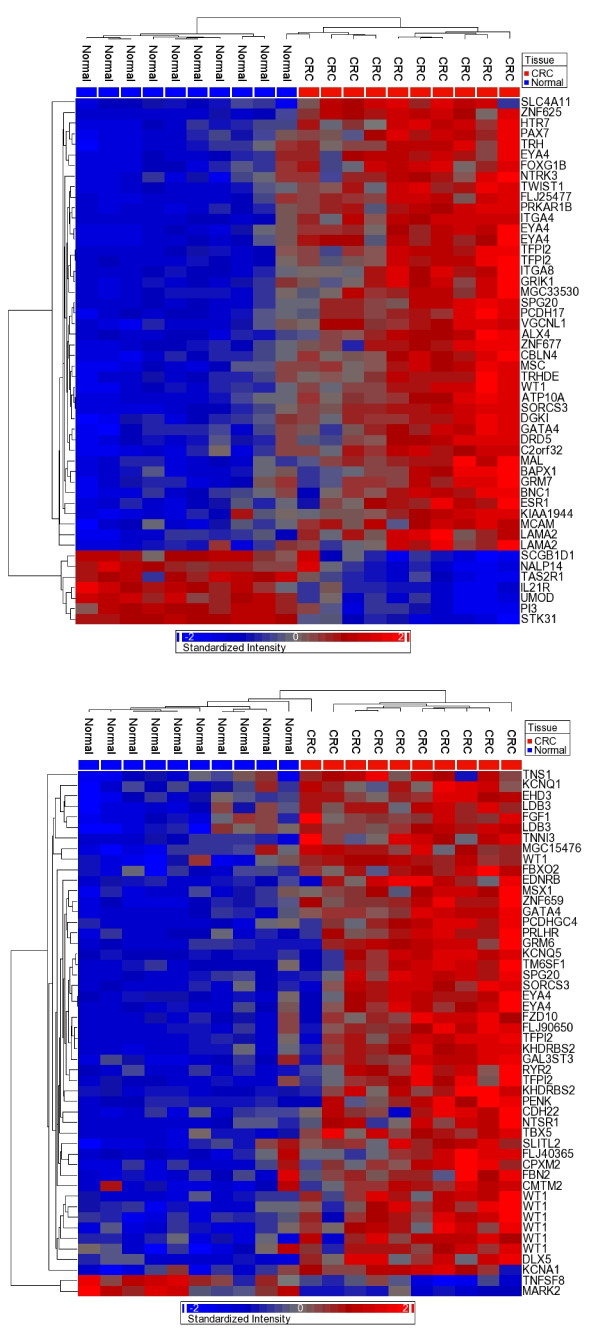
**Unsupervised clustering and heatmap based on the top 50 DML in tumor samples compared to paired normal**. (**A**) The top 50 DML derived from analysis of FF tissue alone. (**B**) The top 50 DML derived from analysis of FFPE tissue alone.

In the next step, we examined whether comparing FFPE tumor samples against FFPE normal samples generates a list of tumor-specific DML that is similar to the list generated from FF tumor and FF normal tissue. In FF samples we identified the DML in CRC using ANOVA after controlling for person-to-person variation, tumor location (left or right colon), sex, and age. In this analysis, a total of 1,404 loci were differentially methylated at p ≤ 0.05 level and Δβ > 0.2; among these loci, 590 passed the criteria of FDR 0.05 and Δβ > 0.2. Hierarchical clustering using the top 50 of these DML (covering 46 genes) is presented in Figure 8A, which shows that the methylation status of these loci was able to effectively separate tumor and normal samples. Similarly, using FFPE (normal and tumor) samples, we also attempted to identify DML using the same ANOVA method. A total of 927 loci were differentially methylated at p ≤ 0.05 level and Δβ > 0.2, but none of them passed the criteria of FDR 0.05 and Δβ > 0.2. However, for the purposes of comparison, we selected the top 50 DML in FFPE (based on unadjusted p-value, covering 40 genes) for unsupervised hierarchical clustering. It was interesting to see that even these genes were able to effectively separate tumor and normal tissue (Figure 8B). When we compared the top 50 DML from FF and the top 50 DML from FFPE samples, there were only 7 loci common to both sets covering six genes: *EYA4, TFPI2, GATA4, SPG20, WT1 *and *SORC53*. This overlap is representative, as the top 100 DML from FF and FFPE samples had 19 loci in common, and the top 30 DML from both sets had 2 loci in common (data not shown). The top 20 DML in FF and FFPE DNA are presented in Additional file 4: Table S1. We also looked at the correlations of the ANOVA p-values of individual DML from FFPE and FF samples. The log_10_-transformed p-values from FF are shown on the x-axis and those from FFPE are shown on the y-axis of the Additional file 5: Figure S4. This also shows lack of strong correlation between them. We also looked at the correlations of β values from paired FF and FFPE samples for a few selected genes e.g. *MGMT, MLH1, VIM, APC*, and *RUNX3*, which are usual suspects in candidate gene approach studies. In general, the result showed suboptimal correlations (see Additional File 6 for all the 49 probes on the chip for these five genes). For example, the chip had a total of 28 loci for *MGMT *gene and the r^2 ^for different loci ranged between 0.000001 and 0.29. For MLH1, however, the r^2 ^was up to 0.68.

**Figure 9:**
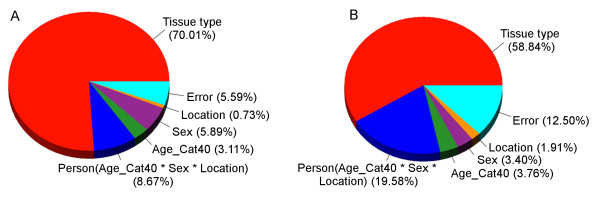
**Partial contribution of the different sources of variation in the methylation data for the top 50 DML derived from (A) FF tissues and (B) FFPE tissues**.

Next, we compared the sources of variation in the top 50 DML from FF and FFPE sample sets (Figure 9). We used the same ANOVA model mentioned above, which factored in person-to-person variation, tissue type (tumor or normal), tumor location (left or right colon), sex, and age. For the top 50 DML in FF samples, tissue type (tumor vs. normal) accounted for a large proportion (70.0%) of the variation in β values. However, for the top 50 DML in FFPE samples, a smaller proportion of variation (58.8%) could be attributed to tissue type. Furthermore, a much larger proportion of variation in FFPE samples was attributed to person-to-person variation (19.6% in FFPE vs. 8.7% in FF).

Our data suggests that data generated by Thirlwell's modified Infinium methylation protocol can identify > 95% loci in FFPE samples, which is significantly fewer than what is seen in FF samples in the unmodified Infinium protocol. Based on these data, it is also possible to separate the tumor and normal samples. In fact, DML sets from FF and FFPE tissue are both effective at differentiating tumor and normal tissue (Figure 8A and 8B). However, these DML sets are very discordant, suggesting that using the modified Infinium assay with FFPE samples may not provide the same biological information as the unmodified assay with FF samples (the gold standard in this case). Furthermore, the DML from FFPE show a greater amount of variation that cannot be attributed to differences in tissue type (Figure 9).

**Figure 10:**
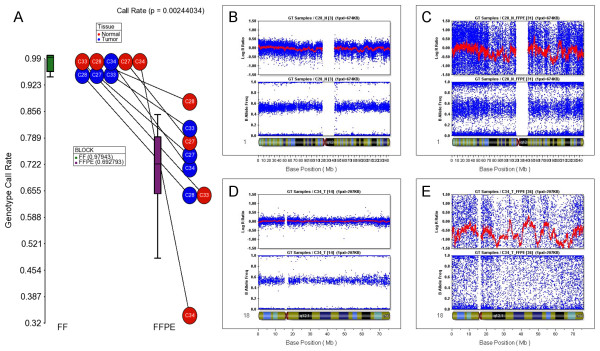
**Infinium genotyping data from paired FF and ligated FFPE samples of CRC and adjacent normal tissue**. These samples are an independent set from those used for Infinium methylation. (**A**) The genotype call rates for ligated FFPE samples were significantly lower and more variable than those of the FF replicates. (**B**) Representative logR ratio (upper pane) and B-allele frequency (lower pane) from chromosome 1 from the FF sample from an individual patient (C_28) (**C**) Corresponding data for the FFPE replicate of the same patient in panel B, who had the higher call rate in FFPE sample. (**D**) Representative logR ratio and B-allele frequency plots from chromosome 18 from the FF sample from a second patient (C_34). (**E**) Corresponding data for the FFPE replicate from the same patient C_34 in panel D, who had lower call rates in FFPE sample.

Finally, we investigated the effect of the additional ligation step for FFPE samples in Illumina's Infinium genotyping assay, which relies on the same chemistry as the methylation assay and can provide independent validation of some of our findings. We compared the performance of the Infinium genotyping assay in ligated FFPE DNA and unmodified FF DNA, using an independent set of 16 DNA samples from 4 patients assayed with Illumina Human Cyto12 SNP Chips. For each patient, we had DNA from tumor and surrounding normal tissue from both FF and FFPE blocks. Ligation was performed on the FFPE samples prior to WGA, as in the modified Infinium Methylation assay. Figure 10A clearly shows that, despite the additional ligation step, the FFPE samples showed significantly lower call rates than corresponding FF samples. The logR ratio and the B-allele frequency plots from representative pairs (Figures 10B-E) also show higher noise and poor performance in ligated FFPE samples. The poor genotype call rates explain at least part of the poor correlation of methylation data in FFPE and FF samples. This further strengthens our conclusion that ligation of FFPE DNA prior to Infinium processing does not make the Infinium chemistry fully compatible with FFPE DNA samples.

## Discussion

Our study has attempted to validate Thirlwell's modified Infinium protocol by using 12 pairs FF and FFPE samples from 12 primary CRC samples and 12 adjacent normal tissues. Thirlwell et al. [30] compared 2 FF ovarian cancer tissue with paired FFPE DNA from ligated and unligated replicates. The authors showed good correlation of β values and intensity between FF and ligated FFPE DNA compared to unligated FFPE DNA. However, Thirlwell did not report changes in differential methylation that are typically investigated in cancer research - for example, whether ligated-FFPE DNA produced the same DML sets as FF DNA, or whether ligated-FFPE DNA had the same power to distinguish tumor samples from normal samples. This kind of hypothesis testing is of particular concern since we have previously reported that in Illumina's DASL whole-genome gene expression microarray, FFPE RNA can yield significantly different results compared to paired FF RNA [31].

In our study, the overall correlation of β values were comparable to Thirlwell's study [30]. When looking at the correlation between FF and FFPE biological replicates, Thirlwell found a median r^2 ^= 0.91, range = 0.88 - 0.96. This was slightly higher than our observed correlations, but not as high as correlations reported by Killian for the GoldenGate assay [23], which ranged from r^2 ^= 0.95 to 0.99, and it may be noted that GoldenGate chemistry is suitable for FFPE samples whereas in principle Infinium chemistry is not. However, our data suggests that correlation is definitely related to the number of data points in the analysis.

Ideally, our experiment would have been designed to include all the four samples from same patient (tumor and normal from FF and FFPE sections) on the same chip to totally eliminate any batch effects. Unfortunately, the FF samples were processed earlier for a separate study. However, in both the cases (FF and FFPE) paired tumor-normal samples were processed on the same chip. Since the β value is calculated from the ratio of the signal intensity values (methylated to total), slight differences in intensities are less likely to affect β.

A few studies have evaluated the use of FFPE tissue was evaluated for lower throughput methylation assays with fewer CpG locations. Balic et al. [27] used HRM to interrogate promoter methylation of two genes (*MGMT *and *APC*), and compared results from paired FFPE and FF samples in 5 human breast cancer cell lines and 3 human prostate cancer cell lines; these results were also validated with the MethyLight qPCR assay. Gagnon et al. validated promoter methylation status of *PLAU *and *TIMP3 *genes in FFPE tissue using methylation sensitive restriction enzyme digestion and qPCR; this was done for paired FFPE and FF samples from 9 primary breast tumor samples and 4 cell line admixtures [20]. Killian et al. evaluated the GoldenGate methylation assay on paired FF and FFPE tissue from 10 lymphoma samples and 10 lymph node hyperplasia samples [23]. They found good correlation of DML between FF and FFPE in different groups, although the number of loci was small. Even though Killian identified lymphoma-specific DML in comparison with hyperplasia samples, the lymphoma and hyperplasia samples were not paired from the same patient (unlike our study, in which tumor and adjacent normal samples were paired).

To our knowledge, our study is unique in addressing this issue at a genome-wide level using a large number of samples and a well-designed experiment to validate tumor-specific DML data derived from paired FFPE DNA (tumor and normal) against DML data from paired corresponding FF tissue DNA. The discrepancy between FFPE and FF samples in our study may reflect: (a) the incompatibility of the Infinium chemistry, including the WGA component; and/or (b) DNA damage induced by tissue fixation, which may lead to misidentification or miscalculation of β values. Fixation-induced changes in methylation status may be ruled out, since previous authors' GoldenGate data and other low-plex methylation data did not find significant differences between FFPE samples and corresponding FF samples.

## Conclusions

In conclusion, ligation-based repair of FFPE DNA may allow the Infinium whole-genome methylation assay to detect a reasonable number of loci, although much fewer than in unmodified FF DNA. Infinium methylation data from ligated FFPE DNA may also differentiate tumor and normal samples, but the DML sets derived from FFPE and FF samples are very discordant and may not provide the same biological information. Therefore, tumor-specific DML identified in FFPE tissue with this method should be interpreted with great caution.

## Competing interests

The authors declare that they have no competing interests.

## Authors' contributions

FJ designed and carried out the genome-wide methylation assay and wrote the manuscript, RR drafted the manuscript, SR processed the tissue samples and carried out the methylation and validation assay, MR and RP collected the tissue samples and did the histopathology, MRZ helped in sample collection and transportation of the samples to USA, RPB and CD helped in methylation microarray, MK organized and supervised the tissue collection and was responsible for histopathology, HA conceived and helped in study design, manuscript preparation, supported and coordinated the study, MGK conceived and designed the study, performed data analysis and wrote the manuscript. All authors read and approved the final manuscript.

## Supplementary Material

Additional file 1 **Figure S1**. Distribution of signal intensities for loci in FF tissue (red) and FFPE tissue (blue). Click here for file

Additional file 2 **Figure S2**. Infinium methylation data from technical replicates. Click here for file

Additional file 3 **Figure S3**. Differentially methylated loci detected by three statistical analyses. Click here for file

Additional file 4 **Table S1**. The top 20 DML derived from multi-way ANOVA of FF and FFPE DNA. Click here for file

Additional file 5 **Figure S4**. Correlation of p-values of DML detected in FF and FFPE tissue using ANOVA. Log_10_-transformed p-values from FF samples are shown on the x-axis, and log_10_-transformed p-values from FFPE samples are shown on the y-axis. Click here for file

Additional file 6 **Table S2**. Correlation of β values from FF and FFPE samples for all the methylation probes for selected five genes. Click here for file
