# Supplementary figures and images for: Interpretation of genome-wide infinium methylation data from ligated DNA in formalin-fixed, paraffin-embedded paired tumor and normal tissue
Source: BMC Res Notes. 2012 Feb 22;5:117. doi: 10.1186/1756-0500-5-117 (PMC3309956; doi:10.1186/1756-0500-5-117)

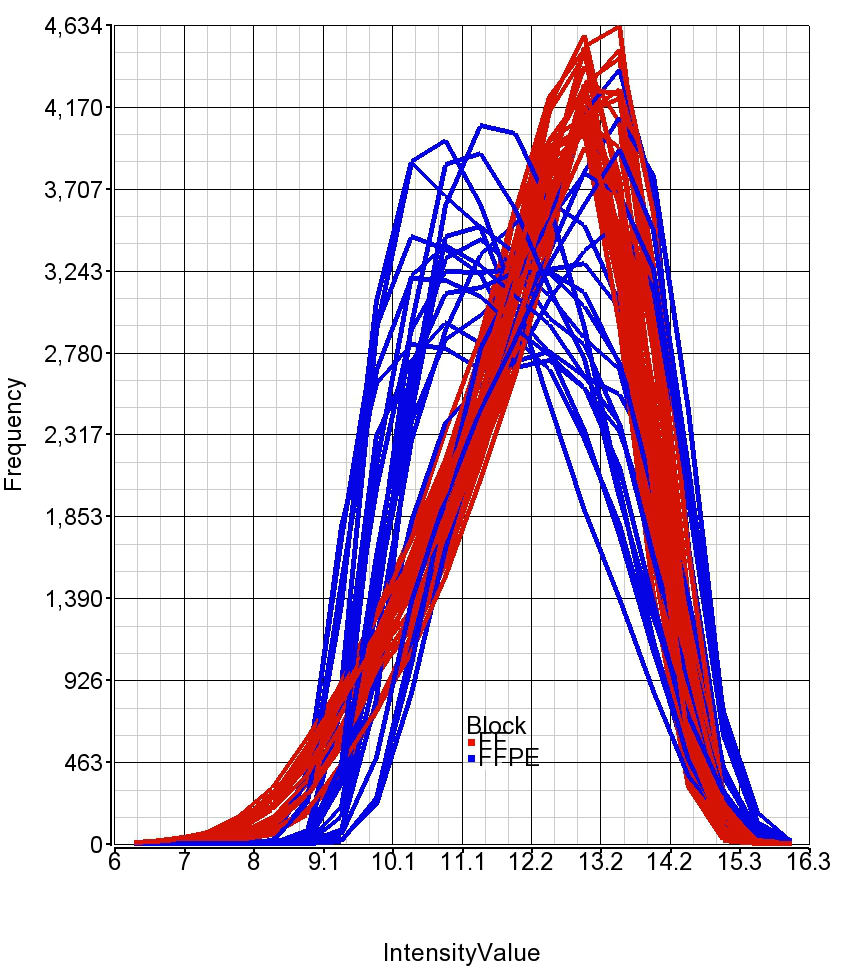

Supplement: Additional file 1 — Figure S1. Distribution of signal intensities for loci in FF tissue (red) and FFPE tissue (blue). [file 1756-0500-5-117-S1.TIFF]

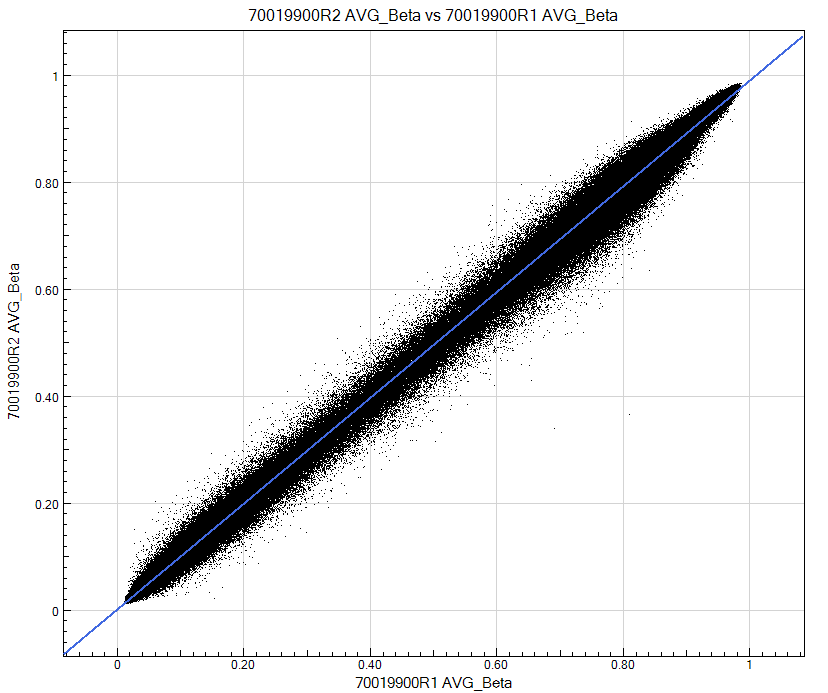

Supplement: Additional file 2 — Figure S2. Infinium methylation data from technical replicates. [file 1756-0500-5-117-S2.TIFF]

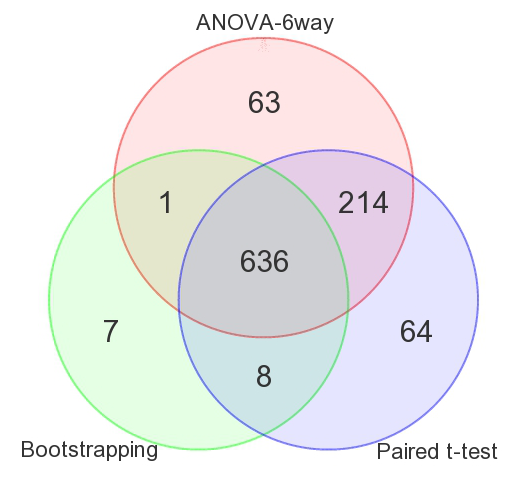

Supplement: Additional file 3 — Figure S3. Differentially methylated loci detected by three statistical analyses. [file 1756-0500-5-117-S3.TIFF]

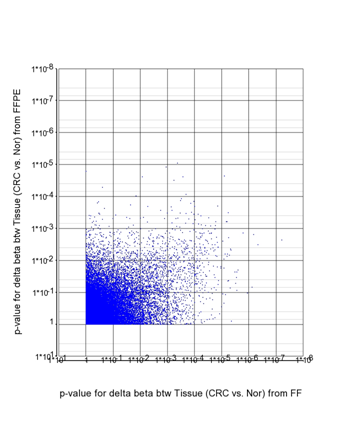

Supplement: Additional file 5 — Figure S4. Correlation of p-values of DML detected in FF and FFPE tissue using ANOVA. Log10-transformed p-values from FF samples are shown on the x-axis, and log10-transformed p-values from FFPE samples are shown on the y-axis. [file 1756-0500-5-117-S5.TIFF]
